# ASO Author Reflections: Prognostic Impact of the Primary Tumor Resection for Lung Cancer Patients Diagnosed with Pleural Dissemination in the Perioperative Period—Importance of Biomarker-Based Treatment Decision Making

**DOI:** 10.1245/s10434-023-13808-6

**Published:** 2023-06-25

**Authors:** Kazuhiko Shien, Toshiya Fujiwara, Shinichi Toyooka

**Affiliations:** 1 Department of Thoracic Surgery, Okayama University Hospital, Okayama, Japan; 2 Okayama University Thoracic Surgery Study Group (OUTSSG), Okayama, Japan; 3 Department of Thoracic Surgery, Hiroshima City Hiroshima Citizens Hospital, Hiroshima, Japan

## Past

Advances in imaging technology, including PET/CT, have improved the accuracy of preoperative staging of non-small-cell lung cancer (NSCLC).^1^ Pleural dissemination (PD) or malignant pleural effusion (MPE) was found during surgery or on postoperative pathology in some patients, although it was not noted on preoperative imaging studies.^2^ According to the eighth edition of the TNM staging system, NSCLC with PD or MPE is classified as stage IVA, and surgical resection is generally not indicated.^3^ Occasionally, primary tumor resection is performed in these patients for histological examination, genetic testing, and symptom relief. Interestingly, some patients with radiologically undetermined PD and/or MPE demonstrated long-term survival, but the prognostic factors remain unknown.

## Present

A review of 9463 NSCLC patients identified 114 cases (1.2%) with PD and/or MPE detected during or after surgery.^4^ The favorable independent prognostic factors for overall survival (OS) were lung adenocarcinoma, clinically undetected lymph node metastasis, and *EGFR* mutation. An analysis of 41 patients with *EGFR* mutations showed significantly better 5-year OS in patients who underwent resection of the primary tumor than those with exploratory thoracotomy. However, no prognostic benefit was observed in patients with wild-type *EGFR* who underwent primary tumor resection as compared with patients who underwent exploratory thoracotomy.

## Future

Biomarker testing is essential for determining the treatment strategy for NSCLC. Recent evidence shows that EGFR tyrosine kinase inhibitors improve the prognosis of advanced NSCLC, and they also function as adjuvant therapy after complete resection. Our study suggests that appropriate biomarker-based patient selection and primary tumor resection may improve the prognosis of patients with stage IVA NSCLC with perioperatively identified PD. A randomized controlled phase III trial is currently examining the therapeutic significance of additional primary tumor resection for stage IVA NSCLC with radiologically undetermined PD (https://jrct.niph.go.jp/en-latest-detail/jRCTs031220666). Further prospective multicenter studies stratified by biomarkers are warranted.
